# Dietary selenium sources alleviate immune challenge induced by *Salmonella Enteritidis* potentially through improving the host immune response and gut microbiota in laying hens

**DOI:** 10.3389/fimmu.2022.928865

**Published:** 2022-08-09

**Authors:** Ruifen Kang, Weihan Wang, Yafei Liu, Shimeng Huang, Jiawei Xu, Lihong Zhao, Jianyun Zhang, Cheng Ji, Zhong Wang, Yanxin Hu, Qiugang Ma

**Affiliations:** ^1^ State Key Laboratory of Animal Nutrition, College of Animal Science and Technology, China Agricultural University, Beijing, China; ^2^ Feed Safety and Healthy Livestock, Beijing Jingwa Agricultural Innovation Center, Beijing, China; ^3^ College of Veterinary Medicine, China Agricultural University, Beijing, China

**Keywords:** selenium, laying hen, *Salmonella Enteritidis*, immune responses, gut microbiota, antioxidant

## Abstract

The aim of this study was to evaluate the effects of different selenium (Se) sources on the immune responses and gut microbiota of laying hens challenged with *Salmonella enteritidis* (*S. Enteritidis*). A total of 240 45-week-old layers were randomly divided into eight groups with six replicates per group according to a 4 × 2 factorial design, including a blank diet without Se supplementation (CON group) and three diets with 0.3 mg/kg Se supplementation from sodium selenite (IS group), yeast Se (YS group), and selenium-enriched yeast culture (SYC group), respectively. After 8 weeks of feeding, half of them were orally challenged with 1.0 ml suspension of 10^9^ colony-forming units per milliliter of *S. Enteritidis* daily for 3 days. The serum was collected on days 3, 7, and 14, and the cecum content was collected on day 14 after challenge. There was no significant difference in laying performance among the eight groups before challenge. The *S. Enteritidis* challenge significantly decreased the laying performance, egg quality, GSH-Px, IgG, and IgM and increased the ratio of feed and egg, malondialdehyde (MDA), *Salmonella*-specific antibody (SA) titers, IL-6, IL-2, IL-1β, and INF-γ. However, SYC increased the level of GSH-Px and IgG and decreased IL-6, while YS decreased the level of IL-2 and IL-1β. What is more, Se supplementation decreased the SA titers to varying degrees and reduced the inflammatory cell infiltration in the lamina propria caused by *S. Enteritidis* infection. In addition, the *S. Enteritidis* challenge disrupted the intestinal flora balance by reducing the abundance of the genera *Clostridium innocuum*, *Lachnospiraceae*, and *Bifidobacterium* and increasing the genera *Butyricimonas* and *Brachyspira*, while Se supplementation increased the gut microbial alpha diversity whether challenged or not. Under the *S. Enteritidis* challenge condition, the alteration of microbial composition by the administration of different Se sources mainly manifested as IS increased the relative abundance of the genera *Lachnospiraceae* and *Christensenellaceae*, YS increased the relative abundance of the genera *Megamonas* and *Sphingomonas*, and SYC increased the genera *Fusobacterium* and *Lactococcus*. The alteration of gut microbial composition had a close relationship with antioxidant or immune response. To summarize, different Se sources can improve the egg quality of layers challenged by *S. Enteritidis* that involves elevating the immunity level and regulating the intestinal microbiota.

## Introduction

*Salmonella enterica serovar* Enteritidis (*S. Enteritidis*) is a gram-negative enteric bacterium that is a major animal-infectious pathogen that can not only cause disease in poultry but also infect humans through the food chain, causing food poisoning and even death (1, 2). *S. Enteritidis* is the most important serotype of *Salmonella*, causing about 40–60% of *Salmonella* infections worldwide (3). Eggs and egg products are the main food carriers for *S. Enteritidis* to spread disease (4, 5). Although the alkaline pH value, high viscosity, and antibacterial protein in albumen create a complex antibacterial environment, *S. Enteritidis* can also be able to resist these stresses and proliferate in eggs, causing food poisoning (6, 7). In 2010, there was an outbreak of *S. Enteritidis* -contaminated eggs in the United States, with as many as 2,752 cases of infection, and more than 500 million defective eggs were recalled (8). Between 2015 and 2018, 16 European countries reported 1,209 large outbreaks of salmonellosis caused by the contaminated eggs of *S. Enteritidis* (9).. What is more, previous studies have found that the *S. Enteritidis* challenge reduced the antioxidant capacity and immune function of laying hens by increasing the serum levels of MDA, IL-1β, and IL-6 (10, 11). Thus, *S. Enteritidis* was a substantial problem for human and animal health, and some strategies are urgently needed to solve this problem.

Selenium (Se) is an essential trace element for the synthesis of some antioxidant enzymes and selenoproteins. It can clean up active oxidative substances in the body and has biological functions such as anti-oxidation, anti-stress, and improving immunity (12–15). Historically, sodium selenite (SS) was the most widely used inorganic Se in animal feed. However, organic Se has higher deposition efficiency and bioavailability, stronger biosafety, and lower toxicity than inorganic Se (16, 17). The sources of organic Se include microorganisms, plants, and animals that absorb inorganic Se and convert it to organic selenium (14, 18, 19). Liao *et al.* compared the effects of dietary supplementation of SS, yeast Se (YS), and selenoprotein on broiler chicks and found that YS was more effective in increasing Se retention in the liver and muscle than IS and selenoprotein (20). Sun *et al.* found that adding 1.0 mg/kg of selenium-enriched earthworms power to laying hens increased the levels of glutathione peroxidase, IgG, and IL-2, further promoting antioxidant activity and immune response (14).

However, there is little information about whether supplementation of different forms of Se could alleviate the adverse effect of laying hens caused by *S. Enteritidis*. The purpose of this experiment was to investigate the effects of dietary supplementation of different Se sources on the performance, immune response, and gut microbiota of laying hens challenged with *S. Enteritidis* to evaluate the effect of different Se sources in resisting the inflammatory response caused by *Salmonella* infection and provide a theoretical basis for Se to defend against *Salmonella* infection in the production practice of laying hens.

## Materials and methods

### Animal experimental ethics

The experiment was allowed by the China Agricultural University Animal Care and Use Committee (A0041011202-1-1, Beijing, China).

### Chemicals and treatments

The common yeast culture and selenium-enriched yeast culture (SYC) used in this experiment were both fermented from the same yeast strain (preservation number: ACCC20060), but with different levels of sodium selenite (Se content was 0 and 30 mg/kg, respectively) in their medium. Both cultures were air-dried at 60°C to inactivate the yeast. Common yeast culture was added to the diet to balance the effect of yeast culture in different treatment diets. The sodium selenite premix (IS), containing 1% of inorganic Se, was purchased from Hebei Yuanda Zhongzheng Biotechnology Co., Ltd. (Hebei, China). The yeast Se (YS), named Alkosel, contains 1,000 mg/kg of organic Se, which was extracted from inactivated whole cell yeast (Lallemand Inc., Montreal, Quebec, Canada). The *Salmonella Enteritidis* (*S. Enteritidis*) strain (preservation number CVCC3377) was purchased from China Institute of Veterinary Drug Control (Beijing, China).

### Animals and experimental design

**Figure 1:**
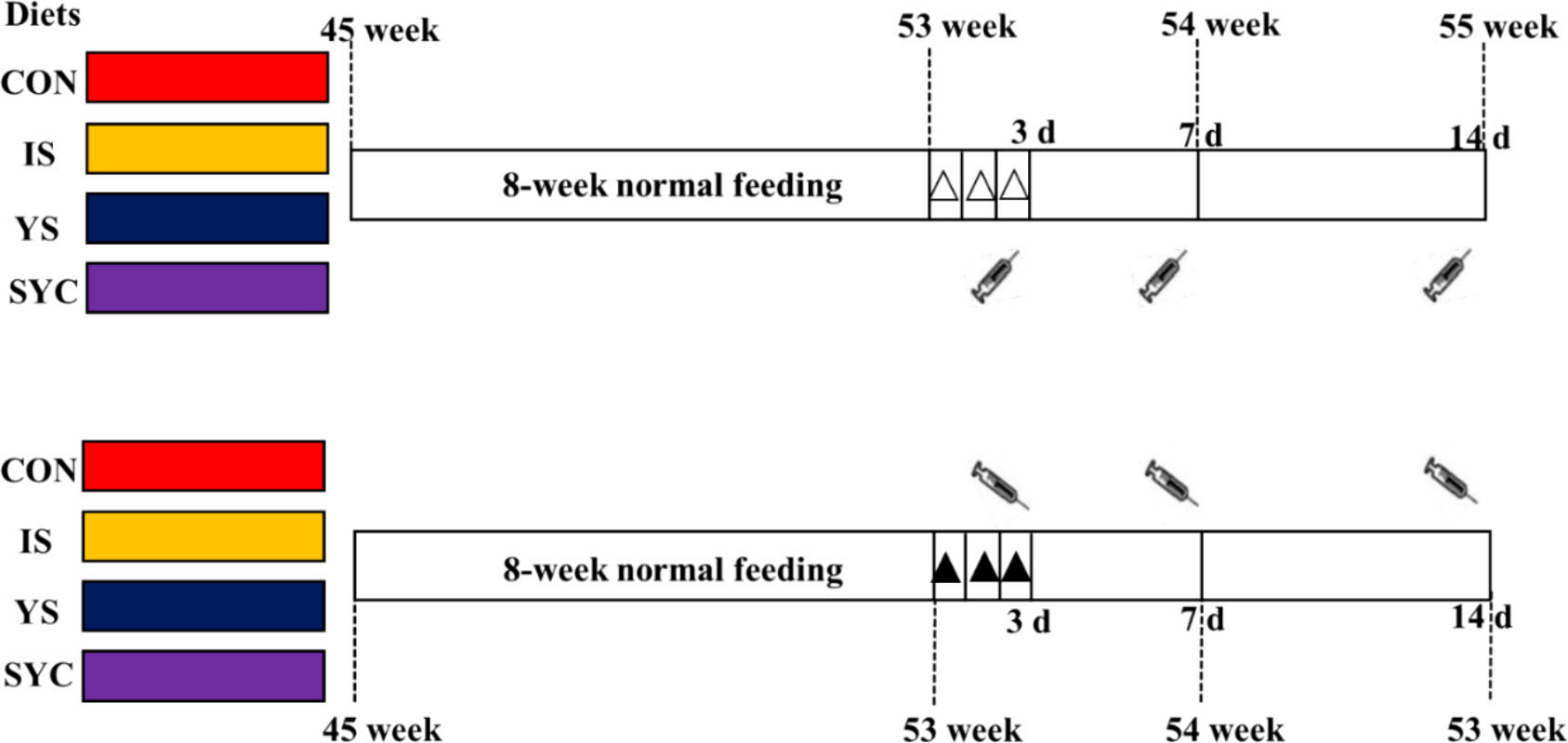
Experimental design for the timeline of supplementation of different Se sources and birds challenged with *S. Enteritidis*.
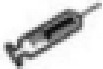
: blood sampling at 3, 7, and 14 days after the challenge with *S. Enteritidis*, △: challenged with physiological saline solution, ▲: challenged with *S. Enteritidis*.

Before the feeding trial, a total of 240 45-week-old laying hens (Peking Pink, Huadu Yukou Poultry Industry Co., Ltd., Beijing) were confirmed as double-negative for *S. Enteritidis* by using PCR method and plate-agglutination assay to test the cloacal swab and serum samples, respectively (21, 22). The birds were randomly divided into eight groups, with six replicates in each group of five birds each, according to a 4 × 2 factorial design. The chickens were housed in wire cages (length, 45 cm × width, 45 cm × height, 45 cm), with one hen per cage, which were equipped with nipple water and a V-shaped feeding trough. The diets of different treatments consisted of a blank diet without Se supplementation (CON group) and three diets with 0.3-mg/kg Se supplementation, which was supplied from sodium selenite (IS group), yeast Se (YS group), and selenium-enriched yeast culture (SYC group), respectively. The whole experimental period consisted of 8 weeks of normal feeding, followed by a 3-day continuous challenge with 1.0 ml suspension of 10^9^ colony-forming units (CFU)/ml (11, 23), or they received the same volume of physiological saline solution (PS), and then the samples were collected at 3, 7, and 14 days after challenge (**Figure 1**). In *S. Enteritidis*, in order to control the horizontal transmission of pathogenic microorganisms, the layer challenged with PS or *S. Enteritidis* was reared respectively in two houses with exactly the same conditions, with four groups of birds in each house. The feed and water were provided *ad libitum*, and the diet composition and nutrient levels are shown in **Supplementary Table S1**.

### Laying performance and egg quality

The egg weight and the number of egg mass were recorded daily based on each replicate. Feed consumption was recorded weekly based on each replicate. The rate of egg production, the mean egg weight, the average feed intake, and the feed/egg ratio were then calculated. At the end of 3, 7, and 14 days after the *S. Enteritidis* challenge, three eggs of each replicate were randomly selected and collected to analyze the egg quality. Egg Haugh units (HU) and egg yolk color were measured by using an egg analyzer (EA-01, Orka Teachnology Ltd., Ramat Hasharon, Israel). The eggshell strength was determined by an egg force reader (EFR-01, Orka Teachnology Ltd., Ramat Hasharon, Israel). The eggshell thickness was determined by a digital egg tester (ESTG-1; Orka Technology Ltd., Ramat Hasharon, Israel)

### Blood collection and serum analysis

At the end of 3, 7, and 14 days after the *S. Enteritidis* challenge, blood samples of five chicken hens in each replicate were collected into heparin treated tubes for 3 h and then centrifuged at 3,000 revolutions per minute for 20 min to get the sera, which were stored at -20°C for further analysis. All samples of five chickens in each replicate were mixed in equal proportions into one sample before analysis (24). The *S. Enteritidis* -specific antibody titer of the serum was determined by using avian *Salmonella* ELISA antibody test kit (catalog number SALS-5P, Biovetest Biotechnology Co., Ltd., Tianjin, China), following the instructions provided by the manufacturer. Serum MDA (catalog number A003-1-2), superoxide dismutase (SOD, catalog number A001-3-2), glutathione peroxidase (GSH-Px, catalog number A005-1-2), immunoglobulin A (IgA, catalog number H108-1-2), immunoglobulin G (IgG, catalog number H106), immunoglobulin M (IgM, catalog number H109), interleukin-1β (IL-1β, catalog number H002), interleukin-2 (IL-2, catalog number H003), interleukin-6 (IL-6, catalog number H007-1-2), and interferon-γ (IFN-γ, catalog number H025) were measured by using corresponding kits (Nanjing Jiancheng Biology Engineering Institute, Nanjing, China) according to instructions.

### Histological examination

At 14 days after *S. Enteritidis* challenge, the duodenum, jejunum, and ileum of chicken were collected and fixed in 4% paraformaldehyde for 24 h. The method of histological examination referred to that in Li *et al.* (25). Images were collected by using the CaseViewer 2.4 software (3DHISTECH Ltd., Budapest, Hungary). The villus height and crypt depth were determined by ImageJ software.

### Immunohistochemistry

The small intestine tissues were paraffin−embedded and cut into slices with a thickness of 4 µm. The slides were dewaxed, dehydrated, and then underwent antigen retrieval. The endogenous peroxidases were blocked with 3% H_2_O_2_ for 15 min at room temperature. The samples were incubated overnight at 4°C with a CD4 mouse-anti-chicken primary antibody (catalog number 8210-26, Southern Biotech; 1:2,000). After three washes, the samples were incubated with a goat-anti-mouse secondary antibody (Beyotime, Beijing, China) for 30 min at 37°C. Visualization was performed by using 3,3′-diaminobenzidine solution. After washing, the tissues were counterstained with hematoxylin (26).

### 16S rRNA gene sequencing

At 14 days after the *S. Enteritidis* challenge, the cecal contents of chicken were collected in tubes and stored at -80°C for further analysis. The cecal contents from five chicken in each replicate were also mixed in equal proportions into one sample (27) to make the microbiome correspond to the phenotypic indicators and serum indices. The total DNA was extracted by using the Omega Bio-tek stool DNA kit (Omega, Norcross, GA, USA) following the manufacturer’s instructions. The V3–V4 region of the 16SrRNA gene was amplified with 338F (5′-ACTCCTACGGGAGGCAGCAG-3′) and 806R (5′-GGACTACHVGGGTWTCTAAT-3′). The PCR products were recovered by 2% agarose gel, purified using AxyPrep DNA Gel Extraction Kit (AxygenBiosciences, Union City, CA, USA), and quantified with QuantiFluor™-ST (Promega, USA). The purified PCR products were sequenced on the Illumina MiSeq PE300 platform (Shanghai MajorBio Biopharma Technology Co., Ltd., Shanghai, China).

### Statistical analysis

The data were analyzed by using GraphPad Prism, version 7.01 (GraphPad Software, Inc., CA, USA). The results were analyzed by two-way ANOVA, followed by Duncan’s multiple comparison when the data were in Gaussian distribution. Otherwise, the Kruskal–Wallis test, followed by Duncan’s multiple comparison, was used for non-normally distributed data. The data were presented as mean ± SEM. *P <*0.05 was considered as significantly different.

The alpha-diversity of the microbiome was calculated by sampling-based OUT analysis by using the MOTHUR program (version v.1.30.1). The beta diversity of the microbiome was displayed by a principal coordinate analysis (PCoA), which was conducted based on the Bray–Curtis distance using QIIME (version 1.17). The difference of bacterial genera that were predominant in bacterial communities among different treatment groups was identified by linear discriminant analysis effect size (LEfSe).

## Results

### Effects of different SE sources on the laying performance of laying hens challenged with *S. Enteritidis*

During the 8-week normal feeding period, no differences of laying performance were observed among the different treatment groups (*P* > 0.05) (**Table 1**). As shown in **Table 2**, the egg production rate and egg mass at 0−3 and 4−7 days were reduced markedly, and the feed-to-egg ratio at 4−7 days was increased after the *S. Enteritidis* challenge (*P* < 0.05). In addition, compared to IS, SYC supplementation significantly increased the egg production rate from 8 to 14 days after the *S. Enteritidis* challenge (*P* < 0.05). There were no differences in the mean weight of eggs and average feed intake of laying hens during 0−3, 4−7, and 8−14 days among different treatment groups (*P* > 0.05).

**Table 1:** Effects of different selenium sources in diets on the performance of layers before challenge by *S. Enteritidis*.

Diets	Houses^a^	Egg production rate(%)	Egg mass(g/day/hen)	Mean weight of eggs(g)	Average feed intake(g)	Feed/egg ratio(g/g)
CON	A	86.67	50.88	58.45	108.92	2.15
CON	B	84.62	49.82	58.87	105.07	2.11
IS	A	86.19	50.06	57.98	105.54	2.12
IS	B	84.05	49.95	59.37	106.52	2.14
YS	A	89.95	51.89	57.98	109.50	2.10
YS	B	88.13	52.91	59.85	110.12	2.10
SYC	A	88.40	51.06	57.56	106.86	2.10
SYC	B	88.97	51.86	58.34	106.81	2.06
SEM		2.069	1.566	0.790	1.962	0.054
CON		85.65	50.35	58.66	107.00	2.13
IS		85.12	50.01	58.67	106.03	2.13
YS		89.04	52.40	58.91	109.81	2.10
SYC		88.69	51.46	57.95	106.84	2.08
SEM		1.463	1.107	0.559	1.387	0.038
	A	87.80	50.97	57.99	107.70	2.12
	B	86.44	51.14	59.11	107.13	2.11
SEM		1.034	0.783	0.395	0.981	0.027
*P*-values	House	0.367	0.885	0.063	0.685	0.733
	Diet	0.168	0.430	0.654	0.275	0.770
	House × diet	0.898	0.906	0.803	0.599	0.908

Different letters indicate statistically signiﬁcant differences among different treatments (P < 0.05).

CON, basal diet; IS, sodium selenite; YS, yeast selenium; SYC, selenium-enriched yeast culture.

a The layers were reared respectively in two houses, with four groups of birds in each house.

**Table 2:** Effects of different selenium sources in diets on the performance of layers challenged by *S. Enteritidis*.

Diets	SE	Egg production rate(%)			Egg mass(g/day/hen)			Mean weight of eggs(g)			Average feed intake(g)			Feed/egg ratio(g/g)		
		0–3 days	4–7 days	8–14 days	0–3 days	4–7 days	8–14 days	0–3 days	4–7 days	8–14 days	0–3 days	4–7 days	8–14 days	0–3 days	4–7 days	8–14 days
CON	–	86.67^ab^	85.83	83.81^ab^	51.08	50.90	48.78	58.91	59.27	58.18	114.90	100.30	98.19	2.26	1.98	2.02
CON	+	85.55^ab^	83.33	83.81^ab^	50.49	48.37	48.41	58.93	58.05	57.77	111.60	103.30	100.20	2.22	2.14	2.07
IS	–	92.22^a^	89.17	80.00^b^	53.45	51.96	46.07	57.99	58.26	57.59	96.89	101.20	98.29	1.82	1.95	2.14
IS	+	82.22^b^	82.50	82.86^ab^	49.21	48.81	48.89	59.84	59.14	59.01	106.00	105.30	97.05	2.15	2.16	1.98
YS	–	90.74^a^	87.69	87.83^ab^	52.55	50.94	50.39	57.91	58.12	57.31	105.53	98.43	101.80	2.01	1.94	2.02
YS	+	88.89^ab^	83.33	85.71^ab^	52.22	49.24	50.06	58.75	59.06	58.40	105.60	105.80	101.20	2.02	2.16	2.02
SYC	–	91.97^a^	91.88	93.92^a^	53.31	52.53	53.87	57.96	57.16	57.35	112.20	99.44	108.30	2.10	1.89	2.01
SYC	+	83.33^b^	84.17	83.81^ab^	48.39	48.90	47.99	58.09	58.12	57.30	98.89	100.70	100.50	2.05	2.06	2.10
SEM		1.535	2.612	2.492	1.309	1.725	1.637	0.778	0.790	0.723	4.729	2.883	3.159	0.117	0.059	0.07
CON		86.11^b^	84.58	83.81^ab^	50.78	49.63	48.60	58.92	58.66	57.97	113.22^a^	101.79	99.19	2.24	2.06	2.05
IS		87.22^ab^	85.83	81.43^b^	51.33	50.39	47.48	58.92	58.70	58.30	101.44^b^	103.25	97.67	1.99	2.05	2.06
YS		89.82^a^	85.51	86.77^ab^	52.39	50.09	50.22	58.33	58.59	57.85	105.54^ab^	102.13	101.52	2.02	2.05	2.02
SYC		87.65^ab^	88.02	88.87^a^	50.85	50.71	50.93	58.02	57.64	57.33	105.55^ab^	100.05	104.38	2.08	1.98	2.06
SEM		1.086	1.847	1.762	0.925	1.22	1.157	0.55	0.559	0.511	3.344	2.038	2.234	0.082	0.042	0.05
	–	90.40^a^	88.64^a^	86.39	52.60^a^	51.58^a^	49.78	58.19	58.20	57.61	107.38	99.82	101.64	2.05	1.94^b^	2.05
	+	85.00^b^	83.33^b^	84.05	50.08^b^	48.83^b^	48.84	58.90	58.59	58.12	105.50	103.79	99.74	2.11	2.13^a^	2.04
	SEM	0.768	1.306	1.246	0.654	0.863	0.818	0.389	0.395	0.362	2.365	1.441	1.580	0.058	0.030	0.035
	SE	< 0.001	0.011	0.202	0.015	0.038	0.428	0.216	0.495	0.332	0.582	0.069	0.408	0.442	< 0.001	0.934
*P*-values	Se	0.148	0.611	0.042	0.600	0.934	0.184	0.590	0.504	0.611	0.133	0.739	0.204	0.171	0.509	0.955
	SE × Se	0.019	0.754	0.097	0.211	0.949	0.102	0.633	0.452	0.544	0.167	0.752	0.476	0.329	0.941	0.361

Different letters indicate statistically signiﬁcant differences among different treatments (P < 0.05).

CON, basal diet; IS, sodium selenite; YS, yeast selenium; SYC, selenium-enriched yeast culture; SE, Salmonella Enteritidis; –, with physiological saline solution challenge; +, with SE challenge.

### Effects of different SE sources on the egg quality of laying hens challenged with *S. Enteritidis*

As demonstrated in **Table 3**, the *S. Enteritidis* challenge had no significant effect on eggshell strength, egg yolk color, egg yolk percent, and eggshell thickness at 3, 7, and 14 days (*P* > 0.05). The egg Haugh unit on day 3 was significantly reduced after the *S. Enteritidis* challenge (*P* < 0.05). However, YS supplementation significantly increased the egg yolk color compared to IS (*P* < 0.05), SYC supplementation significantly increased the egg yolk percent at 7 and 14 days, and IS increased the eggshell thickness at 7 days after the *S. Enteritidis* challenge compared to CON (*P* < 0.05).

**Table 3:** Effects of different selenium sources in diets on the quality of eggs challenged by *S. Enteritidis*.

Diets	SE	Eggshell strength (N)			Egg Haugh unit			Egg yolk color			Egg yolk percent (%)			Eggshell thickness (mm)		
		3 days	7 days	14 days	3 days	7 days	14 days	3 days	7 days	14 days	3 days	7 days	14 days	3 days	7 days	14 days
CON	–	35.69	35.13	30.51	76.64	76.21	76.65	3.67	2.89	3.11	26.57	27.16	26.98	0.39	0.38	0.36
CON	+	32.75	36.07	30.11	74.62	68.60	73.69	3.33	3.00	2.78	26.84	27.43	27.58	0.39	0.39	0.38
IS	–	33.43	32.56	34.06	71.13	75.94	68.47	3.56	3.00	2.95	28.24	26.77	27.68	0.38	0.43	0.38
IS	+	37.60	35.19	29.58	70.32	77.70	69.28	2.67	3.44	3.44	26.83	26.40	26.78	0.40	0.44	0.38
YS	–	35.74	32.80	32.22	74.00	75.67	72.35	3.89	3.89	3.44	27.81	28.04	27.96	0.39	0.42	0.35
YS	+	33.58	30.26	33.75	72.05	75.44	76.44	4.11	3.89	3.56	27.40	27.44	28.08	0.39	0.41	0.39
SYC	–	35.81	26.67	31.85	78.61	72.20	78.01	3.33	3.56	3.00	28.93	29.09	29.16	0.39	0.40	0.36
SYC	+	36.17	34.69	29.77	66.79	73.60	74.18	3.56	3.44	3.11	28.46	28.28	29.49	0.40	0.42	0.37
SEM		2.046	2.316	3.071	2.482	3.759	2.575	0.248	0.373	0.232	0.693	0.617	0.541	0.008	0.014	0.011
CON		34.22	35.60	30.31	75.63	72.41	75.17	3.50^ab^	2.95	2.95	26.70	27.30^b^	27.28^b^	0.39	0.39^b^	0.37
IS		35.52	33.87	31.82	70.73	76.82	68.88	3.11^b^	3.22	3.20	27.54	26.59^b^	27.23^b^	0.39	0.43^a^	0.38
YS		34.66	31.53	32.99	73.03	75.56	74.40	4.00^a^	3.89	3.50	27.61	27.74^ab^	28.02^b^	0.39	0.42^ab^	0.37
SYC		35.99	30.68	30.81	72.70	72.90	76.10	3.44^ab^	3.50	3.06	28.69	28.68^a^	29.33^a^	0.39	0.41^ab^	0.37
SEM		1.447	1.638	2.172	1.755	2.658	1.821	0.176	0.264	0.164	0.490	0.436	0.382	0.006	0.010	0.008
	–	35.17	31.79	32.16	75.10^a^	75.01	73.87	3.61	3.33	3.13	27.89	27.76	27.95	0.39	0.41	0.36
	+	35.03	34.05	30.80	70.95^b^	73.84	73.40	3.42	3.44	3.22	27.38	27.39	27.98	0.40	0.41	0.38
	SEM	1.023	1.158	1.536	1.241	1.879	1.288	0.124	0.186	0.116	0.347	0.309	0.270	0.004	0.007	0.006
	SE	0.924	0.187	0.541	0.031	0.666	0.798	0.288	0.688	0.629	0.321	0.401	0.921	0.320	0.594	0.057
*P*-values	Se	0.819	0.175	0.827	0.303	0.604	0.053	0.021	0.123	0.253	0.075	0.026	0.004	0.929	0.026	0.778
	Se × SE	0.333	0.186	0.793	0.134	0.578	0.417	0.116	0.896	0.539	0.694	0.833	0.542	0.676	0.739	0.401

Different letters indicated statistically signiﬁcant differences among different treatments (P < 0.05).

CON, basal diet; IS, sodium selenite; YS, yeast selenium; SYC, selenium-enriched yeast culture; SE, Salmonella Enteritidis; –, with physiological saline solution challenge; +, with SE challenge.

### Effects of different SE sources on the serum antioxidant status of laying hens challenged with *S. Enteritidis*

**Figure 2:**
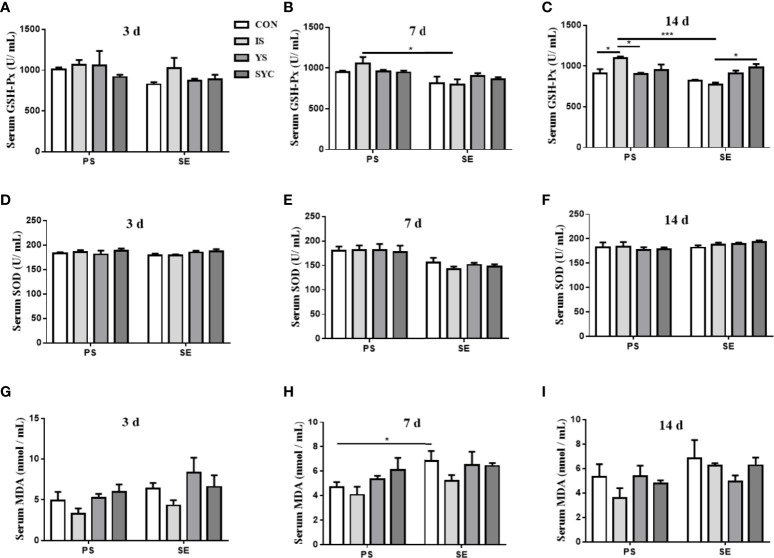
Effects of dietary supplementation with different Se sources on the serum antioxidant status of layers challenged with *S. Enteritidis*. CON, basal diet; IS, sodium selenite; YS, yeast selenium; SYC, selenium-enriched yeast culture; PS, challenged with physiological saline solution; SE, challenged with *S. Enteritidis*. The levels of serum GSH-Px, SOD, and MDA were measured among different periods and treatments **(A–I)**. The data were presented as mean ± SEM. Significance was compared with every other group; *P < 0.05, ***P < 0.001.

As shown in **Figure 2**, the serum GSH-Px at 7 days in the IS group was significantly higher than that in the IS+SE group (*P* < 0.05). The GSH-Px at 14 days in the IS group was significantly higher than that in CON, YS, and IS+SE groups (*P* < 0.05 and *P* < 0.001), and the GSH-Px in the SYC+SE group was significantly higher than that in the IS+SE group (*P* < 0.05). There was no obvious difference in the serum SOD of laying hens among the eight groups at 3, 7, and 14 days after the challenge with PS or *S. Enteritidis* (*P* > 0.05); however, the serum MDA at 7 days in the CON+SE group was significantly increased compared to that in the CON group (*P* < 0.05).

### Effects of different SE sources on serum *Salmonella*-specific antibody titers of laying hens challenged with *S. Enteritidis*

**Figure 3:**
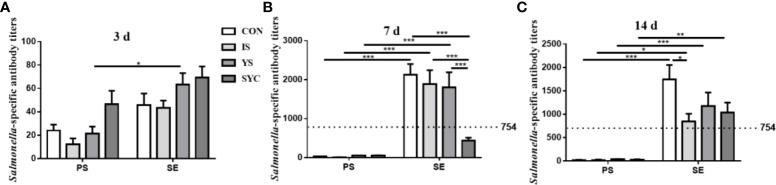
Effects of dietary supplementation with different Se sources on serum *Salmonella*-specific antibody titers of layers challenged with *S. Enteritidis*. CON, basal diet; IS, sodium selenite; YS, yeast selenium; SYC, selenium-enriched yeast culture; PS, challenged with physiological saline solution; SE, challenged with *S. Enteritidis*. The titers were measured among different periods and treatments **(A–C)**. When the antibody titer was >754, the immune status of *Salmonella* is positive, and when the antibody titer was ≤754, the immune status of *Salmonella* is negative. The data were presented as mean ± SEM. Significance was compared with every other group; *P < 0.05, **P < 0.01", ***P < 0.001.

As shown in **Figure 3**, the level of *Salmonella*-specific antibody (SA) titers at 3 days in the YS+SE group was significantly higher than that in the YS group (*P* < 0.05); however, no obvious difference was observed among the other groups (*P* > 0.05). The SA titers at 7 days in the CON+SE, IS+SE, and YS+SE groups were significantly higher than those in the CON, IS, and YS groups (*P* < 0.001), and the titer in SYC+SE was significantly lower than those in the CON+SE, IS+SE, and YS+SE groups (*P* < 0.001). The SA titers at 14 days in the four groups challenged with *S. Enteritidis* were significantly higher than those in the four groups challenged with PS (*P* < 0.05, *P* < 0.01, and *P* < 0.001), and the titers in the IS+SE group were significantly decreased compared to that in the CON+SE group (*P* < 0.05).

### Effects of different SE sources on the immune response of laying hens challenged with *S. Enteritidis*

**Figure 4:**
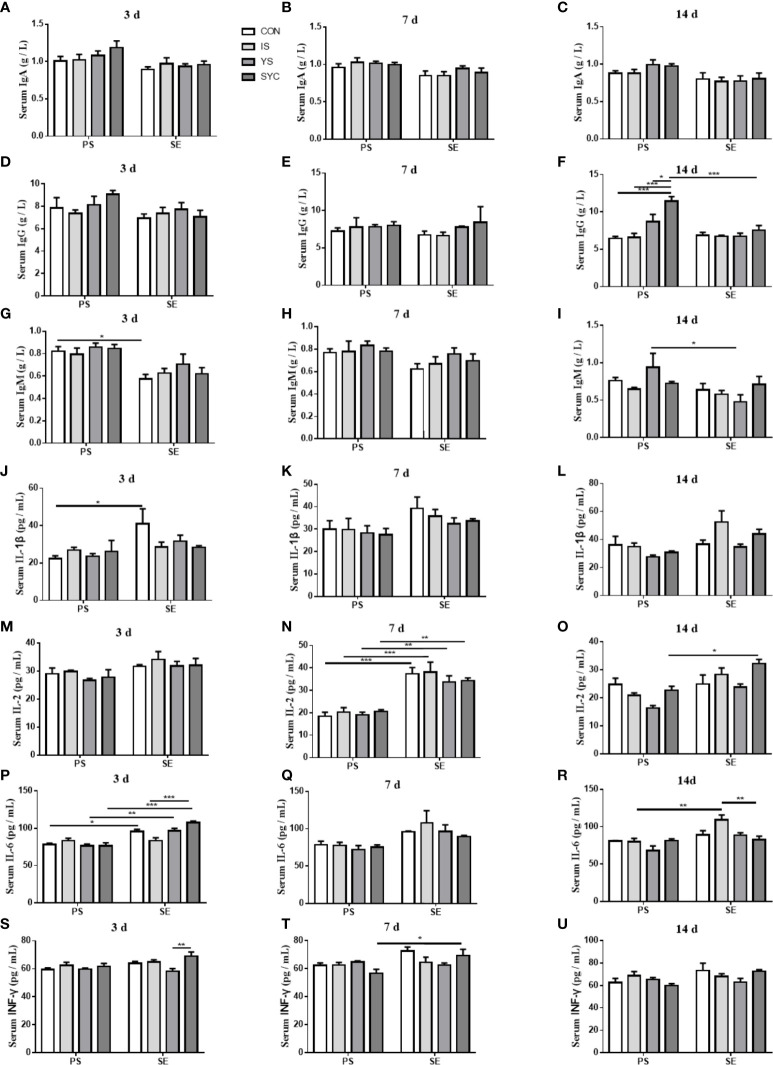
Effects of dietary supplementation with different Se sources on the serum parameters of layers challenged with *S. Enteritidis*. CON, basal diet; IS, sodium selenite; YS, yeast selenium; SYC, selenium-enriched yeast culture; PS, challenged with physiological saline solution; SE, challenged with *S. Enteritidis*. The levels of serum IgA, IgG, IgM, IL-2, Il-6, IL-β, and INF-γ were measured among different periods and treatments **(A–U)**. The data were presented as mean ± SEM. Significance was compared with every other group; *P < 0.05, **P < 0.01, ***P < 0.001.

**Figure 5:**
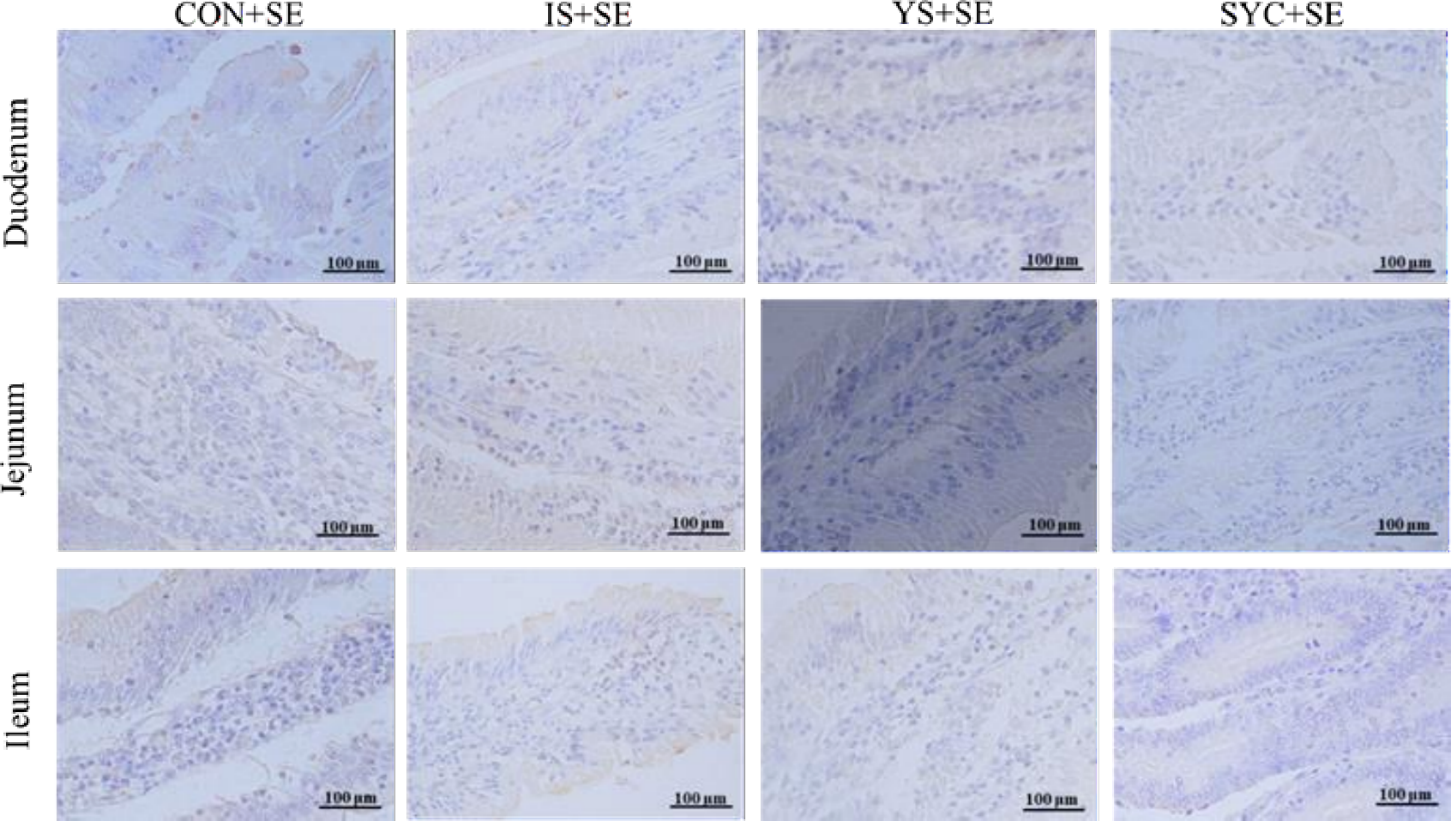
Effects of dietary supplementation with different Se sources on the small intestine immunoexpression of CD4 of laying hens challenged with *S. Enteritidis.* CON, basal diet; IS, sodium selenite; YS, yeast selenium; SYC, selenium-enriched yeast culture; SE, challenged with *S. Enteritidis*. Immunohistochemistry; total magnification ×400.

As shown in **Figure 4**, there was no obvious difference in the serum IgA of laying hens among the eight groups at 3, 7, and 14 days after the challenge with PS or *S. Enteritidis* (*P* > 0.05) (**Figures 4A–C**). The serum IgG in the SYC group was significantly higher than those in the CON, IS, and YS groups (*P* < 0.05). However, compared to SYC, the serum IgG at 14 days was significantly decreased in the SYC+SE group (*P* < 0.05) (**Figure 4F**). The serum IgM at 3 days in CON+SE was significantly decreased compared to that in CON (*P* < 0.05) (**Figure 4G**). The serum IgM at 14 days in YS+SE was significantly decreased compared to that in YS (*P* < 0.05) (**Figure 4I**). The serum IL-1β at 3 days in CON+SE was significantly higher than that in CON (*P* < 0.05) (**Figure 4J**). The serum IL-2 at 7 days in all groups challenged with *S. Enteritidis* was significantly higher than those challenged with PS (*P* < 0.05) (**Figure 4N**); however, only SYC+SE had significantly increased serum IL-2 at 14 days compared to SYC (*P* < 0.05) (**Figure 4O**). The serum IL-6 at 3 days in the CON, YS, and SYC groups of *S. Enteritidis* challenge was significantly higher than those challenged with PS (*P* < 0.05). IL-6 in SYC+SE was also significantly increased compared to that in the IS+SE group (*P* < 0.05) (**Figure 4P**). After the challenge with *S. Enteritidis*, the level of IL-6 at 14 days in IS+SE was significantly higher than those in the IS and SYC+SE groups (*P* < 0.05) (**Figure 4R**). The serum INF-γ at 3 days in SYC+SE was remarkably increased compared to that in the YS+SE group (**Figure 4S**), and the serum INF-γ at 7 days in SYC+SE was significantly higher than that in SYC group (*P* < 0.05) (**Figure 4T**). In addition, *S. Enteritidis* infection caused blue round particles in the lamina propria, which means that the infiltration of inflammatory cells is obvious. Se supplementation reduced the inflammatory cell infiltration in the lamina propria (**Figure 5**).

### Effects of different SE sources on the small intestine morphology of laying hens challenged with *S. Enteritidis*

**Figure 6:**
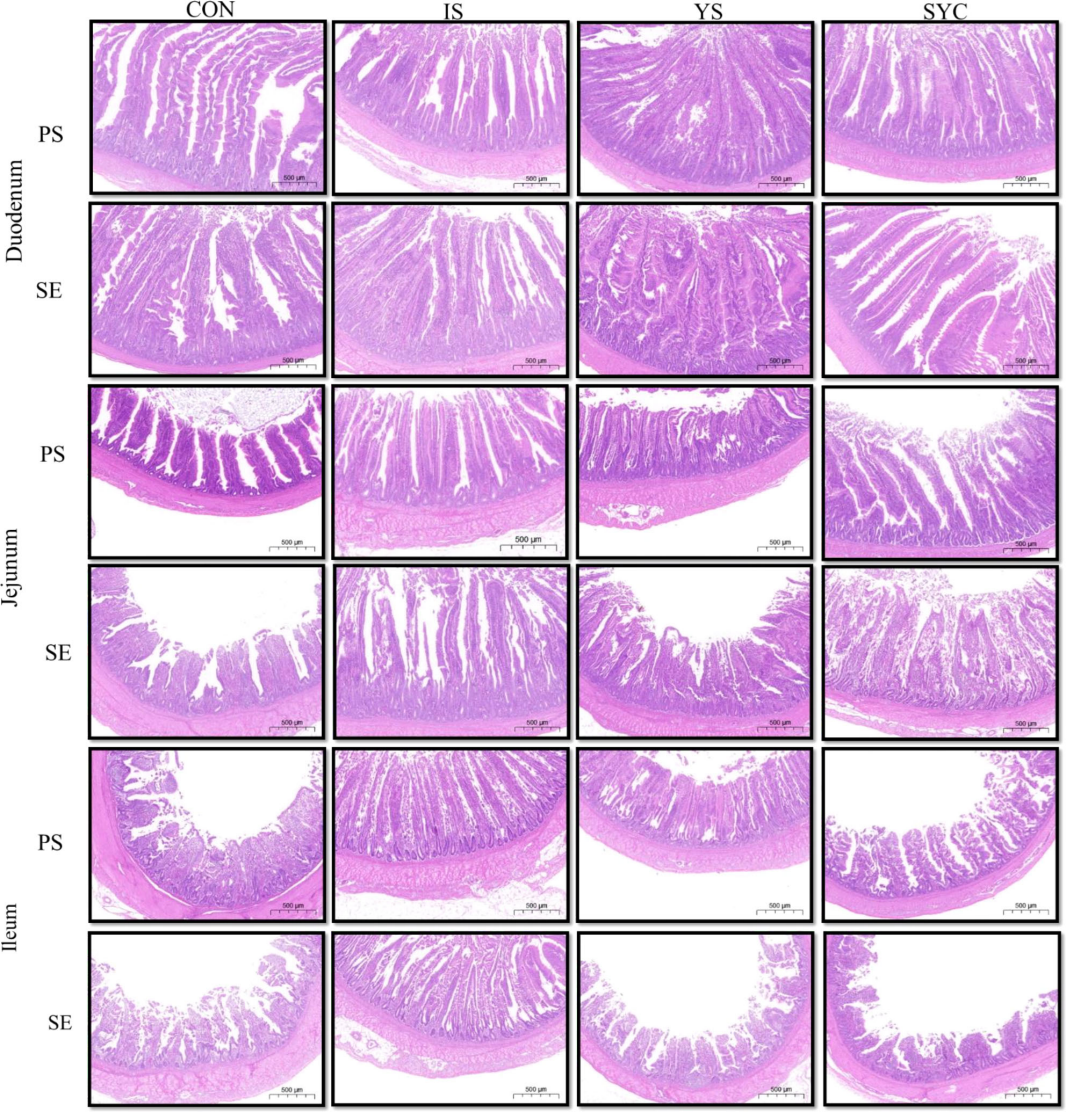
Effects of dietary supplementation with different Se sources on the small intestine histomorphology of laying hens challenged with *S. Enteritidis.* CON, basal diet; IS, sodium selenite; YS, yeast selenium; SYC, selenium-enriched yeast culture; PS, challenged with physiological saline solution; SE, challenged with *S. Enteritidis*. Hematoxylin and eosin staining of the duodenum, jejunum, and ileum of hens (bar = 500 μm).

**Figure 7:**
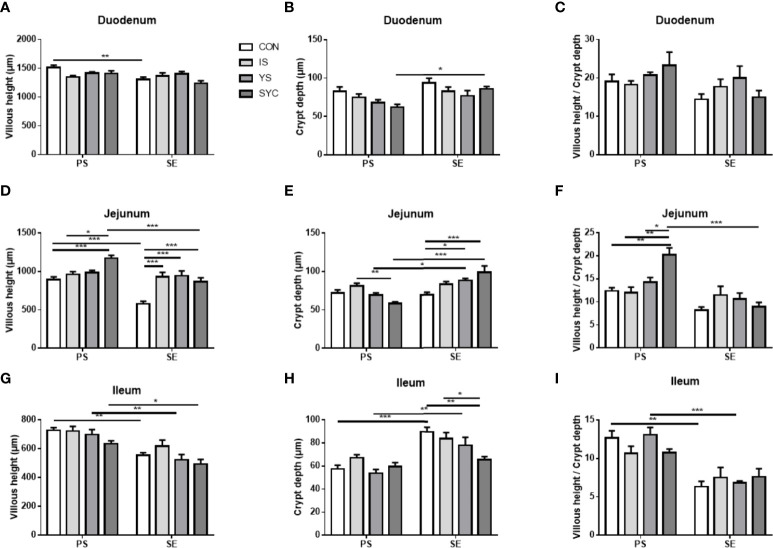
Effects of dietary supplementation with different Se sources on histomorphological measurements in the duodenum, jejunum, and ileum of hens challenged with *S. Enteritidis*. CON, basal diet; IS, sodium selenite; YS, yeast selenium; SYC, selenium-enriched yeast culture; PS, challenged with physiological saline solution; SE, challenged with *S. Enteritidis*. **(A–I)** The villus height, the crypt depth, and the villus/crypt ratio were measured randomly in each sample from different groups. The data were presented as mean ± SEM. Significance was compared with every other group; *P < 0.05, **P < 0.01, ***P < 0.001.

The histopathological changes of the small intestine are shown in **Figure 6** to analyze the effects of Se supplementation on the intestinal morphology of layers after the *S. Enteritidis* challenge. Hematoxylin–eosin (H&E) staining suggested that the morphology of the duodenum, jejunum, and ileum was destroyed by the *S. Enteritidis* challenge, as revealed by crypt atrophy and the adhesion or fusion of villi, whereas Se supplementation could alleviate the degree of intestinal damage caused by the *S. Enteritidis* challenge, which was demonstrated by the increase in villus height and the ratio of villi and crypt while the crypt depth of the duodenum, jejunum, and ileum decreased (*P* < 0.05) (**Figure 7**).

### Effects of different SE sources on the gut microbial composition of laying hens challenged with *S. Enteritidis*

**Figure 8:**
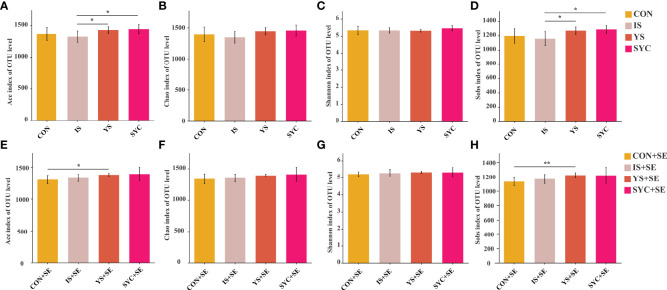
Effects of dietary supplementation with different Se sources on the alpha diversity of the cecal microbiota in layers challenged with *S. Enteritidis*. CON, basal diet; IS, sodium selenite; YS, yeast selenium; SYC, selenium-enriched yeast culture; CON+SE, IS+SE, YS+SE, and SYC+SE mean CON, IS, YS, and SYC challenged with *S. Enteritidis*, respectively. **(A, E)** Ace index of OUT level, **(B, F)** Chao index of OUT level, **(C, G)** Shannon index of OUT level, and **(D, H)** Sobs index of OUT level. The data were presented as means ± SEM. Significance was compared with every other group; *P < 0.05, **P < 0.01.

High-throughput 16S rRNA gene sequencing was conducted to investigate whether Se supplementation would affect the gut microbial composition in laying hens challenged with *S. Enteritidis*. As shown in **Figure 8**, significant differences were observed in alpha diversity among different groups, including Ace and Sobs. Compared to IS, the Ace and Sobs in the YS and SYC groups were significantly increased (*P* < 0.05). The Ace and Sobs in the YS+SE group were significantly higher than that in the CON+SE group (*P* < 0.05).

**Figure 9:**
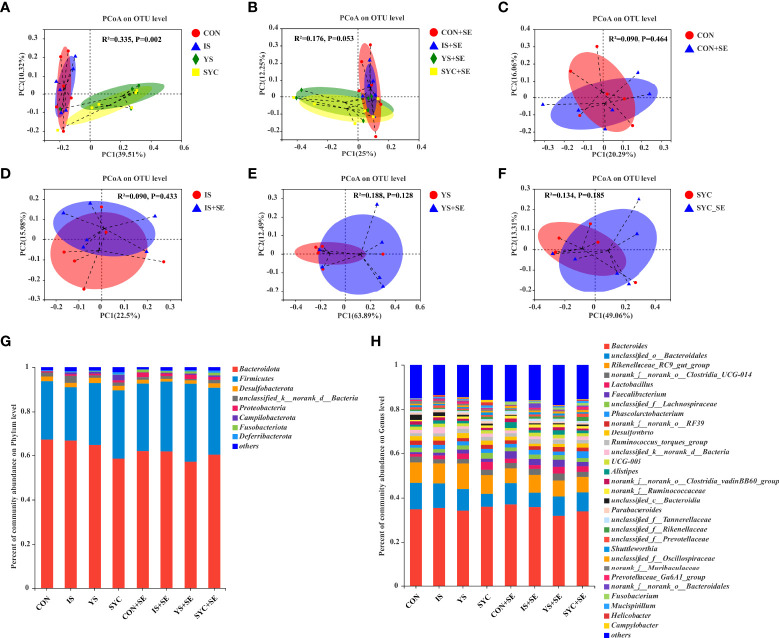
Effects of dietary supplementation with different Se sources on the principal coordinate analysis (PCoA) and the relative abundance in the cecal microbiota of laying hens challenged with *S. Enteritidis*. CON, basal diet; IS, sodium selenite; YS, yeast selenium; SYC, selenium-enriched yeast culture; CON+SE, IS+SE, YS+SE, and SYC+SE mean CON, IS, YS, and SYC challenged with *S. Enteritidis*, respectively. **(A)** PCoA in the CON, IS, YS, and SYC groups. **(B)** PCoA in the CON+SE, IS+SE, YS+SE, and SYC+SE groups. **(C)** PCoA in the CON and CON+SE groups. **(D)** PCoA in the IS and IS+SE groups. **(E)** PCoA in the YS and YS+SE groups. **(F)** PCoA in the SYC and SYC+SE groups. **(G)** Relative abundance of gut microbiota at the phylum level. **(H)** Relative abundance of gut microbiota at the genus level.

A PCoA was conducted to evaluate the differences among different groups. Our results suggested that Se supplementation and *S. Enteritidis* infection would not alter the β diversity of the gut microbial composition (**Figures 9A–F**). The most abundant cecal microbiota composition among different groups was revealed by phylogenetic analysis. At the phylum level, *Bacteroidota*, *Firmicutes*, *Desulfobacterota*, *Proteobacteria*, *Campilobacterota*, *Fusobacteriota*, and *Deferribacterota* were dominant (**Figure 9G**). The predominant genera were *Bacteroides*, *unclassified_o:Bacteroidales*, *Rikenellaceae_RC9_gut_group*, *norank_f:norank_o:Clostridia_UCG-014*, *Lactobacillus*, *Faecalibacterium*, *unclassified_f:Lachnospiraceae*, *Phascolarctobacterium*, *norank_f:norank_o:RF39*, *Desulfovibrio*, *Ruminococcus_torques_group*, *Alistipes*, *Parabacteroides*, and so on (**Figure 9H**).

**Figure 10:**
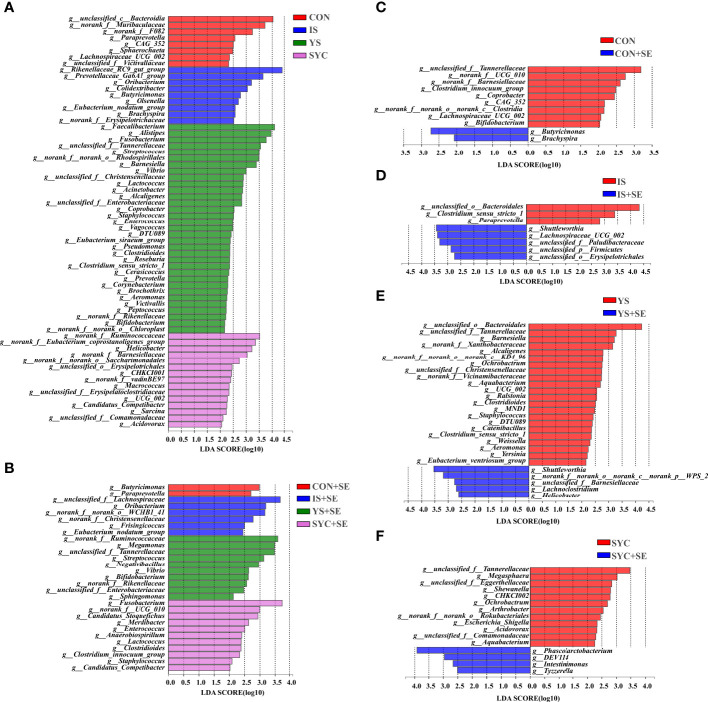
Differentially abundant genera in the gut microbiota of layers among different treatments. CON, basal diet; IS, sodium selenite; YS, yeast selenium; SYC, selenium-enriched yeast culture; CON+SE, IS+SE, YS+SE, and SYC+SE mean CON, IS, YS, and SYC challenged with *S. Enteritidis*, respectively. **(A)** LEfSe analysis of the gut microbiota in the CON, IS, YS, and SYC groups. **(B)** LEfSe analysis of the gut microbiota in the CON+SE, IS+SE, YS+SE, and SYC+SE groups. **(C)** LEfSe analysis of the gut microbiota in the CON and CON+SE groups. **(D)** LEfSe analysis of the gut microbiota in the IS and IS+SE groups. **(E)** LEfSe analysis of the gut microbiota in the YS and YS+SE groups. **(F)** LEfSe analysis of the gut microbiota in the SYC and SYC+SE groups.

As shown in **Figure 10**, the specific bacterial taxa associated with different Se sources and *S. Enteritidis* treatments were identified using LEfSe (LDA score > 2.0). Se supplementation increased the abundance of gut microbial composition before or after the challenge with *S. Enteritidis* (**Figures 10A, B**). Compared to the CON, the relative abundance of *Butyricimonas* and *Brachyspira* was significantly increased, and the relative abundance of *unclassified_f:Tannerellaceae*, *norank_f:UCG_010*, *norank_f:Barnesiellaceae*, *Clostridium_innocuum_group*, *Coprobacter*, *CAG_352*, *norank_f:norank_o:norank_c:Clostridia*, *Lachnospiraceae_UCG_002*, and *Bifidobacterium*, respectively, was significantly decreased in the CON+SE group (**Figure 10C**). The dominant bacteria of the IS group were *unclassified_o:Bacteroidales*, *Clostridium_sensu_stricto_1*, and *Paraprevotella*, while the dominant bacteria in the IS+SE group were *Shuttleworthia*, *Lachnospiraceae_UCG_002*, *unclassified_f:Paludibacteraceae*, *unclassified_p:Firmicutes*, and *unclassified_o:Erysipelotrichales* (**Figure 10D**). The dominant bacteria in the YS group were also *unclassified_o:Bacteroidales*, *unclassified_f:Tannerellaceae*, *Barnesiella*, *Alcaligenes*, *Ochrobactrum*, *Aquabacterium*, *Ralstonia*, and so on, while the dominant bacteria in the YS+SE group were *Shuttleworthia*, *norank_f:norank_o:norank_c:norank_p:WPS_2*, *unclassified_f:Barnesiellaceae*, *Lachnoclostridium*, and *Helicobacter* (**Figure 10E**). In addition, the dominant bacteria in the SYC group were *unclassified_f:Tannerellaceae*, *Megasphaera*, *unclassified_f:Eggerthellaceae*, *Shewanella*, *CHKCI002*, *Ochrobactrum*, *Arthrobacter*, and so on, while the dominant bacteria in the SYC+SE group were *Phascolarctobacterium*, *DEV114*, *Intestinimonas*, and *Tyzzerella* (**Figure 10F**).

### Effects of dietary supplementation with different SE sources on the difference of the gut microbiota and its correlation with the antioxidant and the immunity of laying hens challenged with *S. Enteritidis*

**Figure 11:**
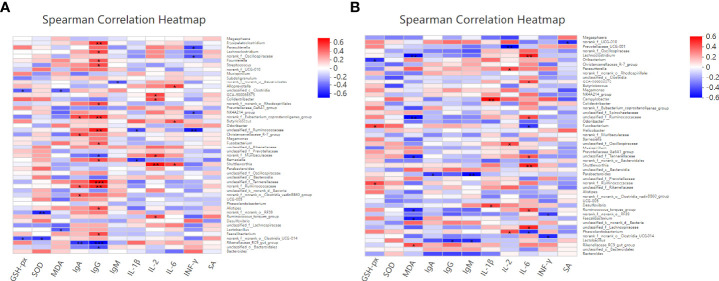
Effects of dietary supplementation with different Se sources on the difference of the gut microbiota and its correlation with the antioxidant and immunity of laying hens at 14 days after the challenge with physiological saline solution **(A)** or *S. Enteritidis* **(B)**. Asterisks indicate significant correlations: *p ≤ 0.05, **p ≤ 0.01, ***p ≤ 0.001. Blue represents a significantly positive correlation (*p* ≤ 0.05), red represents a significantly negative correlation (*p* ≤ 0.05), and white represents no significant correlation (*p* > 0.05). SA, *Salmonella*-specific antibody.

Spearman correlation was performed to predict the correlation among the intestinal microbial communities and the antioxidant and immunity of laying hens 14 days after the challenge with PS or *S. Enteritidis*. As shown in **Figure 11A**, at 14 days after the challenge with PS, *Lactobacillus* was negatively correlated with MDA and *Christensenellaceae_R-7_group* was positively correlated with IgA, but *Rikenellaceae_RC9_gut_group* was negatively correlated with IgA (*P* < 0.05). *Erysipelatoclostridium*, *Lachnoclostridium*, *Fournierella*, *Streptococcus*, *Fusobacterium*, *Barnesiella*, *Alistipes*, and *Faecalibacterium* were positively correlated with IgG (*P* < 0.05), while *Rikenellaceae_RC9_gut_group* was negatively correlated with IgG (*P* < 0.05). *Barnesiella* was positively correlated with IL-1β (*P* < 0.05), *Colidextribacter*, *Shuttleworthia*, and *Ruminococcus_torques_group* were positively correlated with IL-2 (*P* < 0.05), *Alloprevotella*, *Butyricicoccus*, and *Shuttleworthia* were positively correlated with IL-6 (*P* < 0.05), while *Parasutterella* and *NK4A214_group* were negatively correlated with INF-γ (*P* < 0.05).

As shown in **Figure 11B**, at 14 days after the challenge with *S. Enteritidis*, *Fusobacterium* was positively correlated with GSH-Px (*P* < 0.05) and *Ruminococcus_torques_group* and *Faecalibacterium* were negatively correlated with MDA. On the contrary, *Rikenellaceae_RC9_gut_group* was positively correlated with MDA (*P* < 0.05). *Parabacteroides* was negatively correlated with IgA, and *Lactobacillus* was negatively correlated with IgG and IgM (*P* < 0.05). *Campylobacter* and *Desulfovibrio* were positively correlated with IL-1β (*P* < 0.05). *Parasutterella* and *Phascolarctobacterium* were positively correlated with IL-2. On the contrary, *Prevotellaceae_UCG-001* was negatively correlated with IL-2 (*P* < 0.05). *Lachnoclostridium*, *GCA-900066575*, *Shuttleworthia*, and *Ruminococcus_torques_group* were positively correlated with IL-6 (*P* < 0.05), while *Fusobacterium* and *Phascolarctobacterium* were negatively correlated with IL-6 (*P* < 0.05).

## Discussion

*S. Enteritidis* was one of the major factors that affected laying performance for a long time. Previous studies have shown that the *S. Enteritidis* infection of laying hens reduced their feed intake, egg production rate, and body weight (28), which may be related to the colonization of *Salmonella* in the gut (29), disrupting the composition of gut microbiota (30), which, in turn, destroyed the gut barrier function and induced inflammation (31). In addition, oxidative stress is often accompanied by inflammation. When the body was infected by external pathogens, it activated the immune system to clear the infection, and this progress also generated oxidative stress (32). Oxidative stress is mainly manifested as a decrease in antioxidant capacity, such as a decrease in the concentration of antioxidant enzymes such as T-SOD and GSH-PX, an increase in the concentration of MDA, and a further increase in the degree of lipid peroxidation (33). Liu *et al.* reported that *S. Enteritidis* infection significantly increased the level of MDA in the serum of laying hens, further causing oxidative stress (10). Se is an essential trace element and involved in the composition of several metabolic enzymes, such as glutathione peroxidase (GSH-Px) and type I iodothyronine deiodinase (34, 35), and plays a critical role in the application of GSH in resisting the oxidation of host cells (34). A previous study reported that both organic and inorganic Se supplementation could alleviate the heat stress-induced oxidative stress of layers, including increasing the serum concentration of GSH-Px and decreasing the MDA content (15). Se supplementation could increase the effectiveness of immune function through increasing the T cell response, mainly improving IL-2 receptor expression, and prevented immune cells from damage induced by oxidative stress (32). In our study, the *S. Enteritidis* challenge obviously increased the level of MDA, IL-2, IL-6, IL-β, and INF-γ and decreased the level of GSH-Px, IgG, and IgM, further disrupting the intestinal barrier and the balance of the intestinal flora, while Se supplementation alleviated these changes. Therefore, Se supplementation has the potential to be used in alleviating *Salmonella* infection in the production practice of laying hens.

It was worth noting that *Salmonella* infection did not cause changes in the apparent quality and freshness of eggs (4). In the present study, we have found that 10^9^ CFU *S. Enteritidis* challenged for 3 days had no significant effect on the egg quality and laying performance of layers. Fan *et al.* reported that the dietary supplementation of 10^8^ CFU *S. Enteritidis* had no significant effect on the egg quality and production performance of layers, which was consistent with our study. However, it deposited in the tissues and organs of layers, infected the forming eggs, and increased the serum levels of ALT and AST (36). Although *Salmonella* does not affect the performance of birds, the infected *Salmonella* can continue to colonize the cecum and spread to other flocks as they grow (29). Thus, more attention should be paid to the detection of microorganisms in birds to prevent foodborne infections.

CD4 T cells play a critical role in immune protection by recruiting neutrophils, eosinophils, and basophils to the site of infection and responding to a full range of immune responses by producing cytokines and chemokines when the body was infected (37). Previous studies have reported that *Salmonella* infection activated the immune system of the host to conduct a series of immune responses (10, 38). Different cytokines that play important roles in regulating the body’s immune responses resist *Salmonella* infection. The invasion of *Salmonella* onto intestinal epithelial cells caused the secretion of pro-inflammatory cytokines such as IL-6, IL-8, and IFN-γ, which induced systemic inflammation by recruiting immune cells (39, 40). In the present study, systemic inflammation was observed after *S. Enteritidis* infection, including significantly decreased IgM and an increased number of CD4 T cells and the level of IL-1β, IL-2, and IL-6, while IS, YS, and SYC supplementation reversed those changes in IgM, CD4 T cells, and IL-β. SYC also markedly increased the level of IgG compared to CON, IS, and YS. In addition, the level of IL-6 in SYC+SE was significantly higher than that in IS+SE, which is in line with a previous study suggesting that Se supplementation could increase the levels of IgM and IgG of birds, further increasing host immunity (41).

A specific antibody against *Salmonella* plays an important role in host resistance to *Salmonella* infection and directs the clearance of *Salmonella* infection (42). In the present study, we found that *S. Enteritidis* infection significantly increased the specific antibody against *Salmonella* in peripheral serum during the pre-middle period of infection. In line with this outcome, a previous study has reported that *Salmonella* infection can induce high levels of anti-*Salmonella*-specific antibodies in chickens (43). In addition, the present study found that dietary supplementation of organic selenium and inorganic selenium decreased the anti-*Salmonella*-specific antibodies to varying degrees in the middle and late stages of infection. The amount of a specific antibody produced is proportional to the antigen content in the body. Lower levels of a specific antibody in the peripheral serum in the middle and late stages of infection were found in *S. Enteritidis*-infected layers fed with different Se sources, indicating that Se either directly inhibited the growth of *Salmonella* and killed it or SE stimulated the production of anti-*Salmonella*-specific antibodies, further decreasing the load of *Salmonella* in layers. The result suggested that both organic and inorganic Se supplementations could protect against *Salmonella* infection by regulating specific humoral immunity.

As we all know, the small intestine is the main site of nutrient absorption and the body’s first barrier against external substances. It plays a critical role in maintaining gut homeostasis and keeping it healthy (44). Villus height and crypt depth—or the ratio of both (V/C)—were important indicators of intestinal function and maturity. An increase in villus height and V/C ratio indicated a healthy gut and better nutrient absorptive capacity. Conversely, with villus height becoming lower, the intestinal absorptive capacity becomes weaker (45–47). In the present study, *S. Enteritidis* infection significantly destroyed the villi and crypt of the small intestine, which were evidenced by crypt atrophy and villus adhesions. Interestingly, the addition of Se markedly increased the villus height and the ratio of villus and crypt and decreased the crypt depth of the small intestine of laying hens, which further alleviated the damages caused by *S. Enteritidis* infection. Thus, these results suggested that the integrity barrier of the duodenum, jejunum, and ileum of layers was destroyed by *S. Enteritidis* infection, whereas the supplementation of Se alleviated these changes through improving the immune response.

The intestinal flora constituted the intestinal microbial barrier, and a stable intestinal microbial barrier was essential in the digestion and absorption of nutrients and the maintenance of homeostasis in the intestinal environment (48). In the present study, both different organic Se supplementations (YS and SYC) and *S. Enteritidis* infection altered the gut microbial diversity, which was revealed by variations in α diversity, β diversity, and specific bacteria that occurred in different groups. In line with this outcome, a previous study has reported that, in 1-day-old chicks challenged with *Salmonella*, the diversity of the cecal microbiota was markedly decreased (49). Dietary Se supplementation also notably increased the α diversity and β diversity of microbiota in mice (50, 51). The changes of *Salmonella* to the gut microbial composition may be associated with the interaction between pathogen and commensal microbiota or the host mucosal immune response to pathogens or a combination of both of them (52). In addition, according to the LEfSe analyses, the microbial composition of layers was altered by both Se supplementation and *S. Enteritidis* infection. *S. Enteritidis* infection significantly decreased the relative abundance of microbial composition, which indicated that the gut homeostasis was disrupted and certain diseases may occur (53, 54), while Se supplementation reversed these negative effects. *S. Enteritidis* infection also significantly decreased the abundance of *Lachnospiraceae* and *Clostridium*, which could utilize dietary carbohydrate and fiber metabolism to produce butyric acid, regulating both energy metabolism and the immune response of intestinal epithelial cells (55, 56). Butyric acid stimulated the intestinal cells to produce antimicrobial peptide substances that helped to resist the invasion and colonization of *Salmonella*, inhibiting the occurrence of intestinal inflammation and protecting intestinal health (57, 58). In addition, in the present study, YS and SYC supplementation markedly increased the abundance of *Barnesiella*. A previous study has reported that *Barnesiella* was able to clear the intestinal colonization of highly antibiotic-resistant bacteria (59). YS also increased the abundance of *Bacteroidales*, which was considered as an intestinal beneficial bacterial, which can increase immune function and improve intestinal health (60). Collectively, *S. Enteritidis* infection decreased the composition of intestinal microbiota, while Se supplementation could reverse these negative effects by increasing the relative abundance of microbes associated with anti-inflammation, further increasing intestinal homeostasis.

## Conclusion

In conclusion, the present study suggested that selenium (Se) supplementation significantly increased egg production to resist the adverse effects caused by the *S. Enteritidis* challenge. These results also revealed that Se administration could alleviate the intestinal histopathologic damage caused by *S. Enteritidis* infection. In addition, *S. Enteritidis* infection significantly decreased the level of GSH-Px and IgM and increased the level of MDA, IL-1β, and *Salmonella*-specific antibody. However, Se addition reversed these outcomes. Moreover, yeast Se and selenium-enriched yeast culture supplementation maintained intestinal homeostasis through increasing the relative abundance of microbiota related to anti-inflammation, further alleviating the damage caused by *S. Enteritidis* infection.

## Data availability statement

The data presented in the study are deposited in the NCBI repository, and the accession numbers can be found below: https://www.ncbi.nlm.nih.gov/sra/PRJNA849581.

## Ethics statement

The animal study was reviewed and approved by the China Agricultural University Animal Care and Use Committee.

## Author contributions

QM, JZ, CJ, SH, LZ, and ZW designed the study. WW conducted the experiments and collected the data. RK and WW performed the analysis of the experimental data. RK drafted the manuscript and finished the submission. RK, YL, JX and YH detected the samples. All authors contributed to the article and approved the submitted version.

## Funding

This study was supported by the National Key Research and Development Programs of China (2021YFD1300204 and 2021YFD1300405), Special Fund for China Agricultural Research System program (CARS-40-K08), and the National Science Foundation of China (grant no. 31772621).

## Acknowledgments

Special thanks to Professor Dan Liu of China Agricultural University for providing the immunohistochemical kits and to Mr. Yong Zhang of Shijiazhuang Tingrong Technology Co., Ltd. For helping with the emergency allocation of *Salmonella*-specific antibody kits. Without their help, it is impossible for us to complete the supplementary tests in time under the inconvenient condition caused by COVID-19.

## Conflict of interest

The authors declare that the research was conducted in the absence of any commercial or financial relationships that could be construed as a potential conflict of interest.

## Publisher’s note

All claims expressed in this article are solely those of the authors and do not necessarily represent those of their affiliated organizations, or those of the publisher, the editors and the reviewers. Any product that may be evaluated in this article, or claim that may be made by its manufacturer, is not guaranteed or endorsed by the publisher.

## References

[1] Dunkley KD Callaway TR Chalova VI Mcreynolds ME Hume CS Dunkley LF. Foodborne salmonella ecology in the avian gastrointestinal tract. Anaerobe (2009) 15(1-2):26–35. doi: 10.1016/j.anaerobe.2008.05.007 18577459

[2] Howard ZR O'Bryan CA Crandall PG Ricke SC. Salmonella enteritidis in shell eggs: current issues and prospects for control. Food Res Int (2012) 45(2):755–64. doi: 10.1016/j.foodres.2011.04.030

[3] Pearce ME Alikhan NF Dallman TJ Zhou Z Grant K Maiden MCJ. Comparative analysis of core genome MLST and SNP typing within a European salmonella serovar enteritidis outbreak. Int J Food Microbio (2018) 274:1–11. doi: 10.1016/j.ijfoodmicro.2018.02.023 PMC5899760 29574242

[4] Gantois I Ducatelle R Pasmans F Haesebrouck F Gast R Humphrey TJ. Mechanisms of egg contamination by salmonella enteritidis. FEMS Microbiol Rev (2009) 33(4):718–38. doi: 10.1111/j.1574-6976.2008.00161.x 19207743

[5] European Food Safety Authority European Centre for Disease Prevention and Control. The European union summary report on trends and sources of zoonoses, zoonotic agents and food-borne outbreaks in 2011. EFSA J (2013) 11(4):3129. doi: 10.2903/j.efsa.2013.3129

[6] Baron F Nau F Guérin-Dubiard C Bonnassie S Gautier M Andrews SC. Egg white versus salmonella enteritidis! a harsh medium meets a resilient pathogen. Food Microbiol (2016) 53:82–93. doi: 10.1016/j.fm.2015.09.009 26678134

[7] Shen S Fang FC. Integrated stress responses in salmonella. Int J Food Microbiol (2012) 152(3):75–81. doi: 10.1016/j.ijfoodmicro.2011.04.017 21570144 PMC3164900

[8] Zhang W Zheng JX Xu GY. Toward better control of salmonella contamination by taking advantage of the egg's self-defense system: A review. J Food Sci (2011) 76(3):R76–81. doi: 10.1111/j.1750-3841.2011.02053.x 21535852

[9] Pijnacker R Dallman TJ Asl T Hawkins G Larkin L Kotila SM. An international outbreak of salmonella enterica serotype enteritidis linked to eggs from Poland: a microbiological and epidemiological study. Lancet Infect Dis (2019) 19(7):778–86. doi: 10.1016/S1473-3099(19)30047-7 31133519

[10] Liu YJ Zhao LH Mosenthin R Zhang JY Ji C Ma QG. Protective effect of vitamin e on laying performance, antioxidant capacity, and immunity in laying hens challenged with salmonella enteritidis. Poultry Sci (2019) 98(11):5847–54. doi: 10.3382/ps/pez227 31329983

[11] Huang S Rong X Liu M Liang Z Geng Y Wang X. Intestinal mucosal immunity-mediated modulation of the gut microbiome by oral delivery of enterococcus faecium against salmonella enteritidis pathogenesis in a laying hen model. Front Immuno (2022) 13:853954. doi: 10.3389/fimmu.2022.853954 PMC8967290 35371085

[12] Han XJ Qin P Li WX Ma QG Ji C Zhang JY. Effect of sodium selenite and selenium yeast on performance, egg quality, antioxidant capacity, and selenium deposition of laying hens. Poultry Sci (2017) 96(11):3973–80. doi: 10.3382/ps/pex216 29050423

[13] Nawaz F Ashraf MY Ahmad R Waraich EA Shabbir RN. Selenium (Se) regulates seedling growth in wheat under drought stress. Adv Chem (2014) 2014:1–7. doi: 10.1155/2014/143567

[14] Sun X Yue S Qiao Y Sun Z Li H. Dietary supplementation with selenium-enriched earthworm powder improves antioxidative ability and immunity of laying hens. Poultry Sci (2020) 99(11):5344–9. doi: 10.1016/j.psj.2020.07.030 PMC7647737 33142450

[15] Wang W Kang R Liu M Wang Z Zhao L Zhang J. Effects of different selenium sources on the laying performance, egg quality, antioxidant, and immune responses of laying hens under normal and cyclic high temperatures. Animals (2022) 12(8):1006. doi: 10.3390/ani12081006 35454253 PMC9028492

[16] Kim YY Mahan DC. Comparative effects of high dietary levels of organic and inorganic selenium on selenium toxicity of growing-finishing pigs. J Anim Sci (2001) 79(4):942–8. doi: 10.2527/2001.794942x 11325201

[17] Lu J Qu L Ma M Li YF Wang XG Yang Z. Efficacy evaluation of selenium-enriched yeast in laying hens: effects on performance, egg quality, organ development, and selenium deposition. Poultry Sci (2020) 99(11):6267–77. doi: 10.1016/j.psj.2020.07.041 PMC7647803 33142545

[18] Skrivan M Simane J Dlouha G Doucha J. Effect of dietary sodium selenite, Se-enriched yeast and Se-enriched chlorella on egg Se concentration, physical parameters of eggs and laying hen production. Czech J Anim Sci (2006) 51(4):163. doi: 10.1103/PhysRevD.78.034003

[19] Chinrasri O Chantiratikul P Thosaikham W Atiwetin P Chantiratikul A. Effect of selenium-enriched bean sprout and other selenium sources on productivity and selenium concentration in eggs of laying hens. Asian Austral J Anim (2009) 22(12):1661–6. doi: 10.5713/ajas.2009.90220

[20] Liao X Lu L Li S Liu S Zhang L Wang G. Effects of selenium source and level on growth performance, tissue selenium concentrations, antioxidation, and immune functions of heat-stressed broilers. Biol Trace Elem Res (2012) 150(1):158–65. doi: 10.1007/s12011-012-9517-3 23054868

[21] Williams JE Whittemore AD. Serological diagnosis of pullorum disease with the microagglutination system. Appl Microbiol (1971) 21(3):394–9. doi: 10.1128/am.21.3.394-399.1971 PMC377190 5553281

[22] Cohen ND Mcgruder ED Neibergs HL Behle RW Wallis DE Hargis BM. Detection of salmonella enteritidis in feces from poultry using booster polymerase chain reaction and oligonucleotide primers specific for all members of the genus salmonella. Poultry Sci (1994) 73(2):354–7. doi: 10.3382/ps.0730354 8146085

[23] Liu Y Mosenthin R Zhao L Zhang J Ma QG. Vitamin K alleviates bone calcium loss caused by salmonella enteritidis through carboxylation of osteocalcin. J Anim Sci Biotechno (2021) 12(1):1–10. doi: 10.1186/s40104-021-00604-z PMC8276384 34253252

[24] Liao XD Wang G Lu L Zhang LY Lan YX Li SF. Effect of manganese source on manganese absorption and expression of related transporters in the small intestine of broilers. Poultry Sci (2019) 98(10):4994–5004. doi: 10.3382/ps/pez293 31135902

[25] Li R Song M Li Z Li Y Watanabe G Nagaoka K. 4-nitrophenol exposure alters the AhR signaling pathway and related gene expression in the rat liver. J Appl Toxicol (2017) 37(2):150–8. doi: 10.1002/jat.3332 27172127

[26] Stasikowska-Kanicka O Wągrowska-Danilewicz M Danilewicz M. Immunohistochemical analysis of Foxp3+, CD4+, CD8+ cell infiltrates and PD-L1 in oral squamous cell carcinoma. Pathol Oncol Res (2018) 24(3):497–505. doi: 10.1007/s12253-017-0270-y 28669079 PMC5972165

[27] Cao G Zeng X Liu J Yan F Yang C. Change of serum metabolome and cecal microflora in broiler chickens supplemented with grape seed extracts. Front Immuno (2020) 11:610934. doi: 10.3389/fimmu.2020.610934 PMC7753974 33363546

[28] Kulshreshtha G Rathgeber B MacIsaac J Boulianne M Brigitte L Stratton G. Feed supplementation with red seaweeds, chondrus crispus and sarcodiotheca gaudichaudii, reduce salmonella enteritidis in laying hens. Front Microbiol (2017) 8:567. doi: 10.3389/fmicb.2017.00567 28443073 PMC5385333

[29] Gast RK Holt PS. Persistence of salmonella enteritidis from one day of age until maturity in experimentally infected layer chickens. Poultry Sci (1998) 77(12):1759–62. doi: 10.1093/ps/77.12.1759 9872575

[30] Varmuzova K Kubasova T Davidova-Gerzova L Sisak F Havlickova H Sebkova A. Composition of gut microbiota influences resistance of newly hatched chickens to salmonella enteritidis infection. Front Microbiol (2016) 7:957. doi: 10.3389/fmicb.2016.00957 27379083 PMC4911395

[31] Kamada N Seo SU Chen GY Núez G. Role of the gut microbiota in immunity and inflammatory disease. Nat Rev Immunol (2013) 13(5):321–35. doi: 10.1038/nri3430 23618829

[32] McKenzie RC Rafferty TS Beckett GJ. Selenium: an essential element for immune function. Immunol Today (1998) 19(8):342–5. doi: 10.1016/S0167-5699(98)01294-8 9709500

[33] Hou YJ Zhao YY Bo X Cui XS Kim NH Xu YX. Mycotoxin-containing diet causes oxidative stress in the mouse. PloS One (2013) 8(3):e60374. doi: 10.1371/journal.pone.0060374 23555961 PMC3610673

[34] Rotruck JT Pope AL Ganther HE Swanson AB Hoekstra D. Selenium: biochemical role as a component of glutathione peroxidase. Science (1973) 179(4073):588–90. doi: 10.1126/science.179.4073.588 4686466

[35] Berry MJ Banu L Larsen PR. Type I iodothyronine deiodinase is a selenocysteine-containing enzyme. Nature (1991) 349(6308):438–40. doi: 10.1038/349438a0 1825132

[36] Fan S Zheng J Duan Z Xu G. The influences of SE infection on layers’ production performance, egg quality and blood biochemical indicators. J Anim Sci Biotechno (2014) 5(1):1–6. doi: 10.1186/2049-1891-5-4 PMC3898778 24405886

[37] Zhu J Paul WE. CD4 T cells: fates, functions, and faults. Blood (2008) 112(5):1557–69. doi: 10.1182/blood-2008-05-078154 PMC2518872 18725574

[38] Patel S McCormick BA. Mucosal inflammatory response to salmonella typhimurium infection. Front Immuno (2014) 5:311. doi: 10.3389/fimmu.2014.00311 PMC4082011 25071772

[39] McCormick BA Colgan SP Delp-Archer C Miller SI Madara JL. Salmonella typhimurium attachment to human intestinal epithelial monolayers: transcellular signalling to subepithelial neutrophils. J Cell Biol (1993) 123(4):895–907. doi: 10.1083/jcb.123.4.895 8227148 PMC2200157

[40] Gal-Mor O Suez J Elhadad D Porwollik S Rahav G. Molecular and cellular characterization of a salmonella enterica serovar paratyphi a outbreak strain and the human immune response to infection. Clin Vaccine Immunol (2012) 19(2):146–56. doi: 10.1128/CVI.05468-11 PMC3272918 22190395

[41] Cai SJ Wu CX Gong LM Song T Wu H Zhang LY. Effects of nano-selenium on performance, meat quality, immune function, oxidation resistance, and tissue selenium content in broilers. Poultry Sci (2012) 91(10):2532–9. doi: 10.3382/ps.2012-02160 22991539

[42] Beal RK Smith AL. Antibody response to salmonella: its induction and role in protection against avian enteric salmonellosis. Expert Rev Anti-infe (2007) 5(5):873–81. doi: 10.1586/14787210.5.5.873 17914920

[43] Berthelot-Hérault F Mompart F Zygmunt MS Dubray G Duchet-Suchaux M. Antibody responses in the serum and gut of chicken lines differing in cecal carriage of salmonella enteritidis. Vet Immunol Immunop (2003) 96(1-2):43–52. doi: 10.1016/S0165-2427(03)00155-7 14522133

[44] Barker N. Adult intestinal stem cells: critical drivers of epithelial homeostasis and regeneration. Nat Rev Mol Cell Bio (2014) 15(1):19–33. doi: 10.1038/nrm3721 24326621

[45] Hampson DJ. Alterations in piglet small intestinal structure at weaning. Res Vet Sci (1986) 40(1):32–40. doi: 10.1016/S0034-5288(18)30482-X 3704321

[46] Qin SM Zhang KY Ding XM Bai SP Zeng QF. Effect of dietary graded resistant potato starch levels on growth performance, plasma cytokines concentration, and intestinal health in meat ducks. Poultry Sci (2019) 98(9):3523–32. doi: 10.3382/ps/pez186 31329991

[47] Turner JR. Intestinal mucosal barrier function in health and disease. Nat Rev Immunol (2009) 9(11):799–809. doi: 10.1038/nri2653 19855405

[48] Bourlioux P Koletzko B Guarner F Braesco V. The intestine and its microflora are partners for the protection of the host: report on the danone symposium “The intelligent intestine,” held in Paris, June 14, 2002. Am J Clin Nutr (2003) 78(4):675–83. doi: 10.1093/ajcn/78.4.675 14522724

[49] Mon K Perot S Halstead MM Chanthavixay G Chang HC Garas L. Salmonella enterica serovars enteritidis infection alters the indigenous microbiota diversity in young layer chicks. Front Vet Sci (2015) 2:61. doi: 10.3389/fvets.2015.00061 26664988 PMC4672283

[50] Kasaikina MV Kravtsova MA Lee BC Seravalli J Peterson DA Walter J. Dietary selenium affects host selenoproteome expression by influencing the gut microbiota. FASEB J (2011) 25(7):2492–9. doi: 10.1096/fj.11-181990 PMC3114522 21493887

[51] Zhu H Lu C Gao F Qian Z Yin Y Kan S. Selenium-enriched bifidobacterium longum DD98 attenuates irinotecan-induced intestinal and hepatic toxicity *in vitro* and *in vivo* . BioMed Pharmacother (2021) 143:112192. doi: 10.1016/j.biopha.2021.112192 34649340

[52] Barman M Unold D Shifley K Amir E Hung K Bos N. Enteric salmonellosis disrupts the microbial ecology of the murine gastrointestinal tract. Infect Immun (2008) 76(3):907–15. doi: 10.1128/IAI.01432-07 PMC2258829 18160481

[53] Manichanh C Rigottier-Gois L Bonnaud E Gloux K Pelletier E Frangeul L. Reduced diversity of faecal microbiota in crohn’s disease revealed by a metagenomic approach. Gut (2006) 55(2):205–11. doi: 10.1136/gut.2005.073817 PMC1856500 16188921

[54] Guo F Geng Y Abbas W Zhen W Wang S Huang Y. Vitamin D3 nutritional status affects gut health of salmonella-challenged laying hens. Front Nutr (2022) 9. doi: 10.3389/fnut.2022.888580 PMC9127613 35619956

[55] Donohoe DR Garge N Zhang X Sun W O'Connell TM Bunger MK. The microbiome and butyrate regulate energy metabolism and autophagy in the mammalian colon. Cell Metab (2011) 13(5):517–26. doi: 10.1016/j.cmet.2011.02.018 PMC3099420 21531334

[56] Xu B Yan Y Yin B Zhang L Qin W Niu Y. Dietary glycyl-glutamine supplementation ameliorates intestinal integrity, inflammatory response, and oxidative status in association with the gut microbiota in LPS-challenged piglets. Food Funct (2021) 12(8):3539–51. doi: 10.1039/D0FO03080E 33900316

[57] Van Immerseel F Boyen F Gantois I Timbermont L Bohez L Pasmans F. Supplementation of coated butyric acid in the feed reduces colonization and shedding of salmonella in poultry. Poultry Sci (2005) 84(12):1851–6. doi: 10.1093/ps/84.12.1851 16479940

[58] Fernández-Rubio C Ordonez C Abad-González J Garcia-Gallego A Honrubia MP Mallo JJ. Butyric acid-based feed additives help protect broiler chickens from salmonella enteritidis infection. Poultry Sci (2009) 88(5):943–8. doi: 10.3382/ps.2008-00484 19359681

[59] Ubeda C Bucci V Caballero S Djukovic A Toussaint NC Equinda M. Intestinal microbiota containing barnesiella species cures vancomycin-resistant enterococcus faecium colonization. Infect Immun (2013) 81(3):965–73. doi: 10.1128/IAI.01197-12 PMC3584866 23319552

[60] Delday M Mulder I Logan ET Grant G. Bacteroides thetaiotaomicron ameliorates colon inflammation in preclinical models of crohn’s disease. Inflammation Bowel Dis (2019) 25(1):85–96. doi: 10.1093/ibd/izy281 PMC6290787 30215718

