# Supplementary material for: Dietary selenium sources alleviate immune challenge induced by Salmonella Enteritidis potentially through improving the host immune response and gut microbiota in laying hens
Source: Front Immunol. 2022 Aug 9;13:928865. doi: 10.3389/fimmu.2022.928865 (PMC9396296; doi:10.3389/fimmu.2022.928865)
Supplement: Supplementary file 1 [file Table_1.docx]

**Table S1. Diet composition and nutritional levels**.

|  | **CON** | **IS** | | **YS** | **SYC** |
| --- | --- | --- | --- | --- | --- |
| **Ingredients** | **Composition (%)** | | | | |
| Corn | 63.93 | 63.93 | | 63.93 | 63.93 |
| Soybean meal | 23.60 | 23.60 | | 23.60 | 23.60 |
| Common yeast culture | 1.00 | 1.00 | | 1.00 | 0.00 |
| Selenium-enriched yeast culture | 0.00 | 0.00 | | 0.00 | 1.00 |
| Limestone | 9.00 | 9.00 | | 9.00 | 9.00 |
| Dicalcium Phosphate | 1.60 | 1.60 | | 1.60 | 1.60 |
| Salt | 0.30 | 0.30 | | 0.30 | 0.30 |
| DL-methionine | 0.11 | 0.11 | | 0.11 | 0.11 |
| L-lysine hydrochloride | 0.08 | 0.08 | | 0.08 | 0.08 |
| Threonine | 0.02 | 0.02 | | 0.02 | 0.02 |
| Tryptophan | 0.02 | 0.02 | | 0.02 | 0.02 |
| Vitamins ^1^ | 0.04 | 0.04 | | 0.04 | 0.04 |
| Trace minerals ^2^ | 0.30 | 0.30 | | 0.30 | 0.30 |
| Nutrient | Levels | | | | |
| ME^3^, MJ/kg | 14.91 | | 14.91 | 14.91 | 14.91 |
| Crude protein, % | 15.40 | | 15.40 | 15.40 | 15.40 |
| Calcium, % | 3.64 | | 3.64 | 3.64 | 3.64 |
| Phosphorus, % | 0.35 | | 0.35 | 0.35 | 0.35 |
| Lysine, % | 0.81 | | 0.81 | 0.81 | 0.81 |
| Total sulfur amino acids % | 0.58 | | 0.58 | 0.58 | 0.58 |
| Threonine, % | 0.61 | | 0.61 | 0.61 | 0.61 |
| Selenium^4^, mg/kg | 0.049 | | 0.352 | 0.373 | 0.368 |

CON: basal diet, IS: sodium selenite, YS: yeast selenium, SYC: selenium-enriched yeast culture.

^1^The vitamins provided per kg of diet: vitamin A, 8000 IU; vitamin D3, 3600 IU; vitamin E, 21 IU; vitamin K3, 4.2 mg; vitamin B1, 3 mg; vitamin B2, 10.2 mg; folic acid, 0.9 mg; calcium pantothenate, 15 mg; smoke Acid, 45 mg; vitamin B6, 5.4 mg; vitamin B12, 0.024 mg; biotin, 0.15 mg. ^2^ The trace minerals provided per kg of diet: iron, 60 mg; manganese, 60 mg; copper, 8 mg; zinc, 80 mg; iodine, 0.35 mg. ^3^ ME: metabolizable energy. ^4^ Except for the selenium content, all others are calculated values.
